# Genome-wide analysis of the *Hsp70* gene family in rice reveals that *OsHsp70-9* plays a significant role in heat stress response

**DOI:** 10.1186/s12870-025-07975-9

**Published:** 2025-12-28

**Authors:** You Zhou, Wenhao Lv, Manqiong Zhu, Chunni Wang, Linjun Yu, Yaling Bao, Chenghang Tang, Meng Zhang, Qiang Huang, Dongxia Ye, Yu Lin, Yingyao Shi

**Affiliations:** 1 Bio-breeding Laboratory of Anhui Province, School of Agronomy of Anhui Agricultural University, Hefei, 230036 China; 2 Rice Reserach Institute of Uzbekistan, Urtachirchiq District, Sholikor Satllment, Madaniyat Street, Tashkent Region, Uzbekistan

## Abstract

Heat shock protein 70 (Hsp70) is a key molecular chaperone in plant stress responses playing a crucial role in maintaining growth homeostasis under heat stress. In this study, we revealed the evolutionary conservation of the *OsHsp70* family through phylogenetic tree construction, collinearity analysis, and Ka/Ks analysis. We also screened the heat-tolerance candidate genes using various methods, including tissue-specific expression analysis, expression profiling under heat stress, haplotype analysis of heat-tolerance indicators in 620 rice germplasms, amino acid site mutation, and RT-qPCR. The results showed that the promoter regions of *Hsp70* genes contained multiple *cis*-acting elements related to stress responses. Seven pairs of intragenic collinear genes were identified in *OsHsp70s*, and Ka/Ks analysis indicated that this family has undergone strong purifying selection. Expression analysis showed that most *OsHsp70* genes have tissue-specific expression. Haplotype analysis indicated that the Hap2 of *OsHsp70-9* plays an important role in the heat-stress tolerance of rice, and a non-synonymous mutation of *OsHsp70-9* led to changes in the domain. RT-qPCR analysis showed that the expression of *OsHsp70-9* varied significantly under heat stress, which is consistent with the expression profiling results under heat stress. Overall, this study demonstrates that *Hsp70* genes play a potential role in the stress tolerance of rice, and provides important clues for better understanding the molecular mechanisms underlying the adaptation of rice to environmental changes and the adaptive evolution of *Hsp70* genes.

**Supplementary Information:**

The online version contains supplementary material available at 10.1186/s12870-025-07975-9.

## Introduction

Rice is a crucial global food source that feeds more than 50% of the world’s population [2, 10]. Heat stress triggered by global warming is an important environmental factor that restricts rice growth and yield [55]. It has been estimated that the global population will exceed 10 billion by 2050, and 10% of the population will still be faced with food shortages at that time. Agricultural production decline caused by global warming has already posed serious threats to food security. Therefore, it is highly necessary to breed heat-tolerant crops to ensure a stable and sufficient food supply [1, 23, 32]. Heat stress affects different growth stages of rice, specifically including the germination, seedling, tillering, booting, heading, flowering, and grain-filling stages [14, 27, 48, 50]. Heat stress adversely affects the photosynthetic capacity, increases the level of reactive oxygen species (ROS), causes protein denaturation, and triggers metabolic imbalance, ultimately impairing carbon dioxide (CO₂) fixation, pollen/tube growth, pollen/grain viability, and the processes of pollination and fertilization [34, 37].

Heat stress induced by heat-shock treatment activates the endogenous protein defense network in plants, significantly upregulating the expression of heat-shock proteins (Hsps), which are key components in the cellular stress-response mechanism [28]. Based on their molecular weight, Hsps can be divided into five major categories, including Hsp100, Hsp90, Hsp70, Hsp60, and small-molecular-weight Hsps [44]. Among these Hsp families, Hsp70 is known as a major Hsp family. It consists of two functional domains, namely the nucleotide-binding domain (NBD) and the substrate-binding domain (SBD) [7]. Hsp70 is of great significance in plant growth, development, and stress response [42]. Particularly, it plays a crucial role in maintaining growth homeostasis of plants under heat stress at sub-lethal temperatures [33, 36, 40]. Under heat stress, it acts as a molecular chaperone, protecting plants by assisting the restoration of correct folding of proteins and repair of denatured proteins [13]. There have been reports on the genome-wide identification of the Hsp70 gene family in soybean (*Glycine max*), potato (*Solanum tuberosum*), tobacco (*Nicotiana tabacum*), maize (*Zea mays*) and apple (*Malus domestica*) [15, 21, 25, 39, 52]. This gene family has also been intensively studied in plants such as rice (*Oryza sativa*), pepper (*Capsicum annuum*), and Arabidopsis [3, 12, 16]. In Arabidopsis, overexpression, knockout, or knockdown of a single *Hsp70* gene caused no obvious phenotypic changes. However, the double-mutant *hsp70-1/hsp70-4* and triple-mutant *hsp70-2/hsp70-4/hsp70-5* plants showed defective developmental phenotypes, including a shortened specific growth period, curled and round-shaped leaves, twisted petioles, thin stems, and short siliques [18]. Kim et al. found that the thermosensitive mutants carrying a T-DNA insertion in *OsHsp70CP1* developed severe chlorosis in the seedling leaves when grown at a constant high temperature (40 °C), while plants grown at a constant 27 °C exhibited a normal phenotype [17]. Qi et al. discovered that overexpression of *mtHsp70* inhibited heat- and H₂O₂-induced programmed cell death (PCD) in rice protoplasts, as reflected by higher cell viability, reduced DNA laddering, and chromatin condensation [31].

Although the *Hsp70* gene family has been studied in various species, there has been little research on the impact of *Hsp70* haplotypes on phenotypes in rice under heat stress. This study employed bioinformatic methods to identify and analyze the expression patterns of the rice *Hsp70* gene family under heat stress. The key candidate genes (*OsHsp70-9*) were screened through a haplotype analysis and gene expression profiling. The findings are expected to improve our understanding of rice tolerance to abiotic stresses and provide valuable resources for future rice genetic improvement and molecular-assisted breeding.

## Results

### Identification and phylogenetic analysis of *OsHsp70* members

**Fig. 1:**
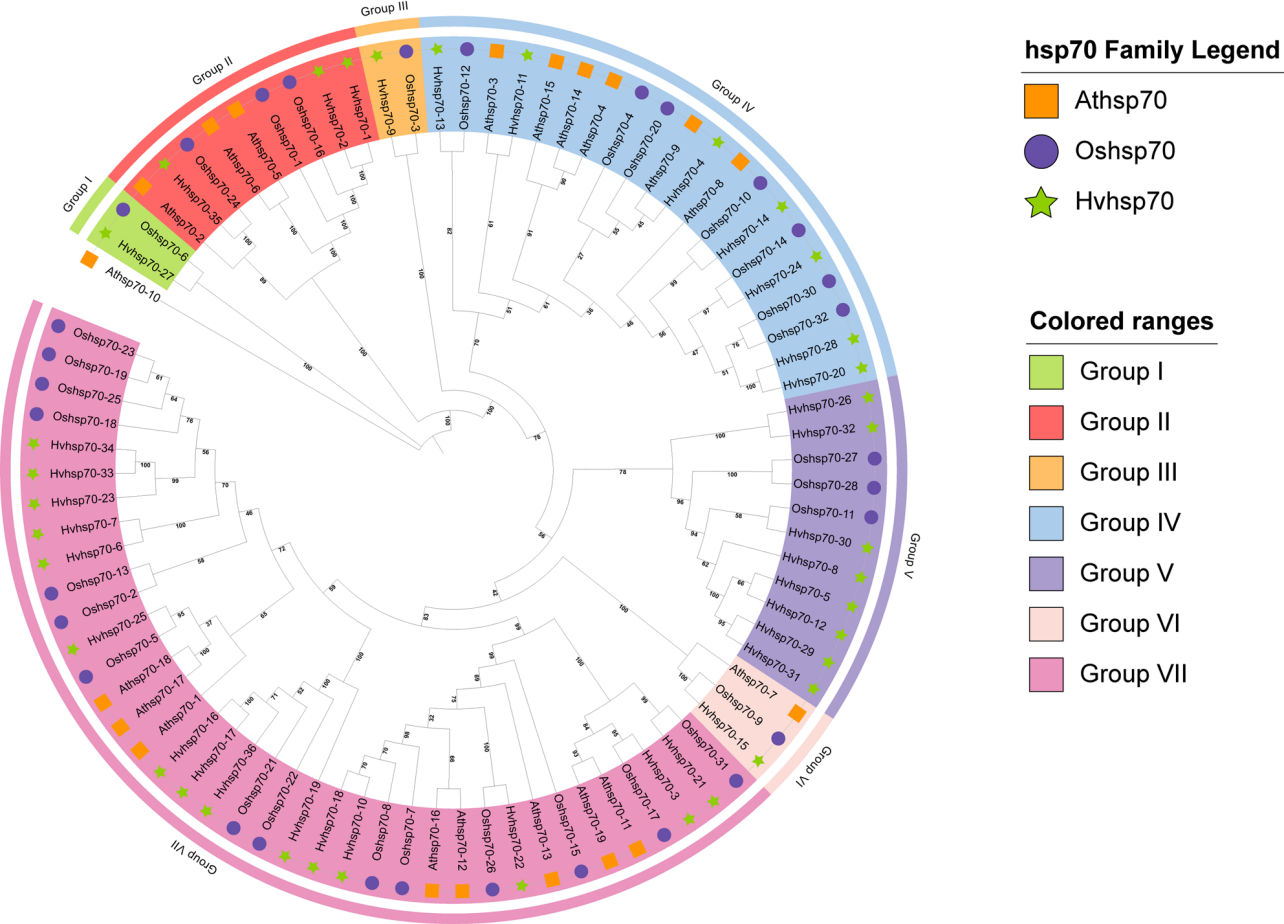
Phylogenetic analysis of OsHsp70, AtHsp70, and HvHsp70 members. Different colors represent different sub-families

Through retrieval of conserved domains using the Hidden Markov Model (HMM) and the Basic Local Alignment Search Tool (BLAST), we identified a total of 32 Hsp70 proteins in the japonica rice genome (Nipponbare) (Table S1).The chromosomal localization map of OsHSP70 is presented in Figure S1. We employed the same method to identify the heat-shock protein 70 (Hsp70) families in Arabidopsis thaliana, a model crop for dicotyledonous plants, and Hordeum vulgare, an important crop within the Poaceae family. Multiple sequence alignments were performed on 19 AtHsp70 proteins, 36 HvHsp70 proteins, and 32 OsHsp70 proteins, and a phylogenetic tree was constructed using TBtools software (Fig. 1). Based on the branches of the tree, the Hsp70 members were divided into seven subgroups. Group 1 and Group 3 had the fewest members. Group 1 contained only 1 HvHsp70 member and 1 OsHsp70 member, while Group 7 had the most members, including 8 AtHsp70 members, 15 HvHsp70 members, and 15 OsHsp70 members. Group I、Group III and Group V included no AtHsp70 members, indicating that the genes in this subgroup emerged after the divergence of dicotyledonous and monocotyledonous plants during plant evolution. As depicted in Figure S1, the motif composition of OsHsp70 genes within a given phylogenetic subgroup exhibits relative conservation. For example, the majority of members in Group IV possess core motifs including Motif 3, Motif 8, Motif 4, Motif 2, and Motif 9, with a fixed arrangement order. Notably, there exist distinct disparities in motifs among different subgroups. In comparison to Group IV, Group I and Group II are deficient in Motif5 and Motif7. These dissimilarities not only mirror the evolutionary conservation of functions within subgroups but also the functional specialization among them (Figure S2).

### Promoter *cis*-acting element profiling of *OsHsp70* members

**Fig. 2:**
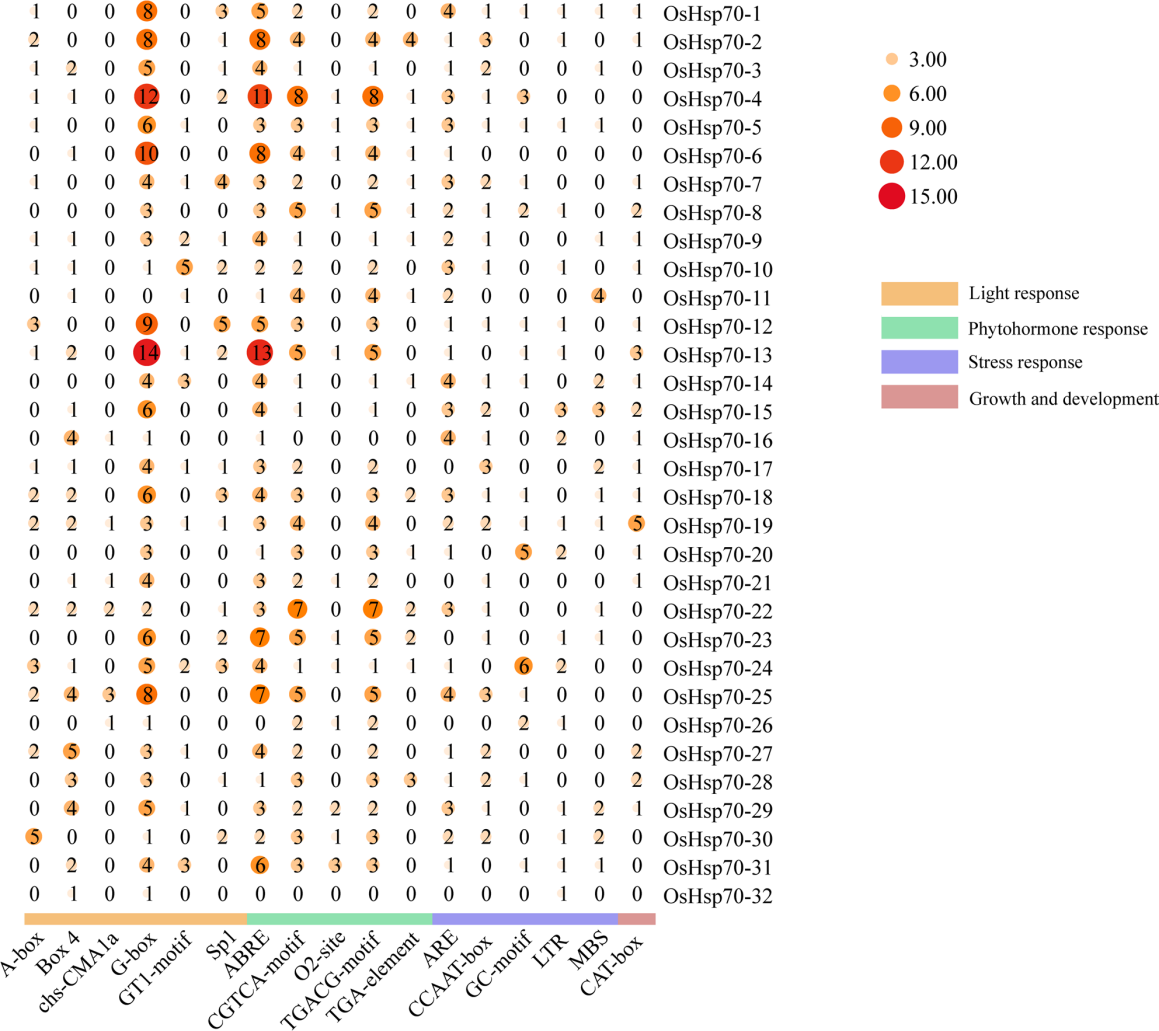
Analysis of *cis*-acting elements of *OsHsp70* genes. Heatmap analysis of *cis*-acting elements in the promoter regions of *OsHsp70* genes. In the heatmap, the values represent the number of different *cis*-acting elements, with a darker color indicating a larger number

The *cis*-acting element analysis was carried out on the 2000-bp sequence upstream of the *OsHsp70* genes using the online tool PlantCARE. As shown in Fig. 2, among the *cis*-acting elements related to light response, the G-box element was the most abundant, which occurred 153 times and accounted for 52.04% of the total. The ABRE element was the most abundant *cis*-acting element related to plant hormone response, which occurred for 130 times and accounted for 36.72% of the total. The ARE element was the most frequent *cis*-acting element related to stress response, accounting for 34.68% of the total number of elements. The CAT-box was the most common *cis*-acting element associated with growth and development. The enrichment of ABA-responsive elements (ABRE) implies that OsHsp70 might be activated via the ABA hormone signaling pathway. The enrichment of stress-responsive key elements (ARE) indicates its participation in regulating oxidative stress associated with heat stress. These two classes of elements collectively confer upon OsHsp70 the capacity for coordinated regulation in hormonal regulation and heat-stress response.

### Collinearity analysis of *Hsp70s*

**Fig. 3:**
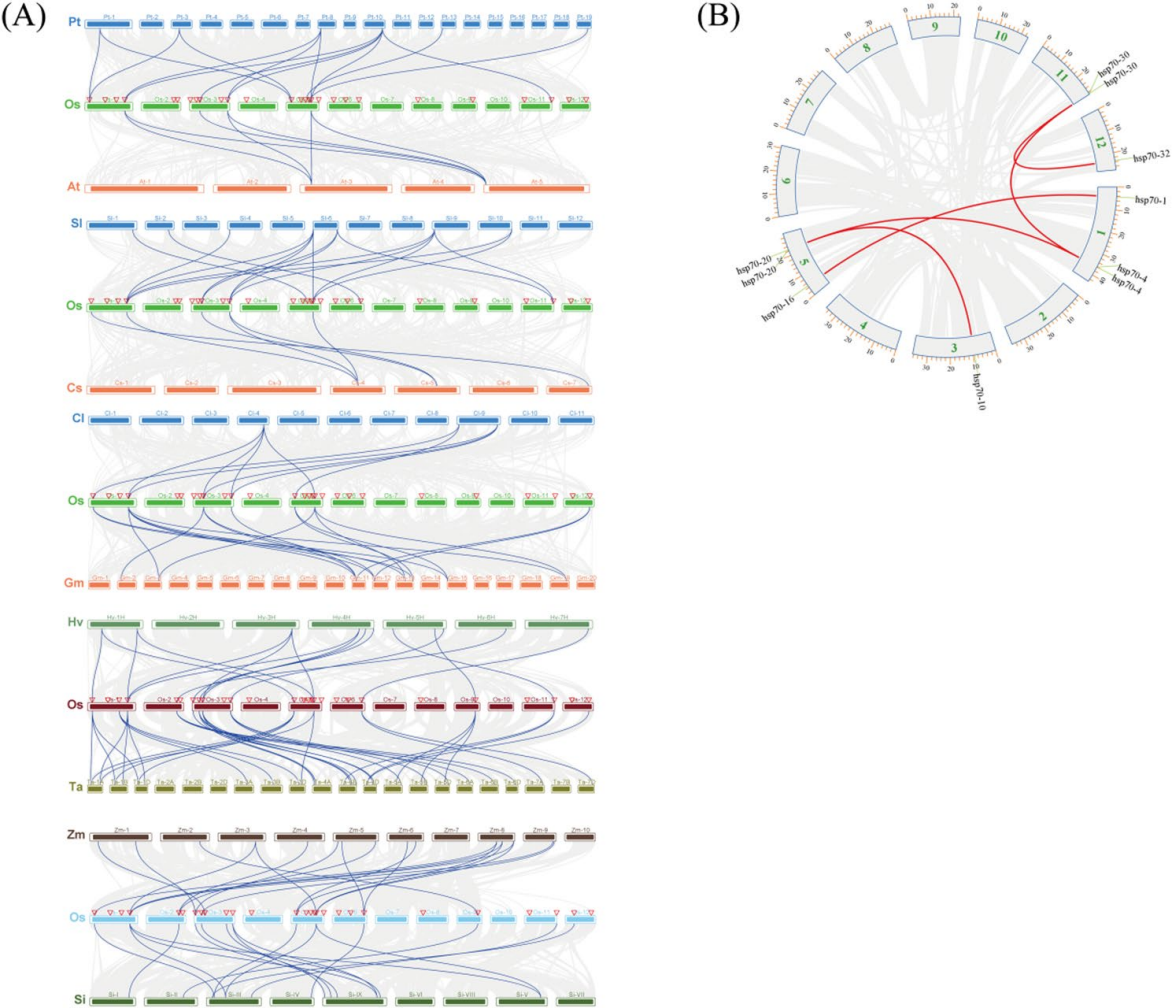
Collinearity analysis of *Hsp70* genes. **A** Collinearity analysis of *Hsp70* genes among *Oryza sativa*, *Populus trichocarpa*,* Arabidopsis thaliana*,* Solanum lycopersicum*,* Cucumis sativus*,* Citrullus lanatus*,* Glycine max*,* Hordeum vulgare*,* Triticum aestivum*,* Zea mays*, and *Setaria italica*. **B** Collinearity analysis of *OsHsp70* genes within rice

To clarify the evolutionary relationships of *Hsp70s*, a collinearity analysis was conducted using the MCScanX software, which involved *Oryza sativa*,* Populus trichocarpa*,* Arabidopsis thaliana*,* Solanum lycopersicum*,* Cucumis sativus*,* Citrullus lanatus*,* Glycine max*,* Hordeum vulgare*,* Triticum aestivum*,* Zea mays*, and *Setaria italica* (Fig. 3). Rice had 14, 6, 15, 6, 9 and 17 collinear gene pairs with the dicotyledonous plants of *P. trichocarpa*,* A. thaliana*,* S. lycopersicum*,* C. sativus*,* C. lanatus*, and *G. max*, while 16, 35, 18, and 17 collinear gene pairs with the monocotyledonous plants of *H. vulgare*,* T. aestivum*,* Z. mays*, and *S. italica*, respectively. These results indicated that rice has more collinear genes with monocotyledonous plants than with dicotyledonous plants. This suggests that the Hsp70 gene family originated prior to the divergence of monocots and dicots. Following their divergence, the two plant lineages underwent specific amplification and functional divergence, each building upon the ancestral Hsp70 gene family. The collinearity results within species showed that there were five pairs of collinear genes within *OsHsp70s*. Tandem duplication is defined as the continuous arrangement of homologous genes at the same chromosomal locus. In contrast, segmental duplication pertains to the situation where homologous genes are situated at distinct positions on either the same or non - homologous chromosomes, and they are discontinuously distributed. The findings presented in Fig. 3B demonstrate that the collinear genes within the OsHsp70 gene family all stem from segmental duplications. This suggests that the evolution and expansion of this family are predominantly driven by genomic segment rearrangement. To further explore the influence of selection pressure on the evolution of the OsHsp70 family, we calculated the non-synonymous substitution rate (Ka), synonymous substitution rate (Ks), and Ka/Ks ratio of the homologous genes in the OsHsp70 family. As shown in Table 1, the Ka/Ks ratios for the five pairs of genes (hsp70-20/hsp70-10, hsp70-20/hsp70-4, hsp70-16/hsp70-1, hsp70-30/hsp70-4, and hsp70-30/hsp70-32) are 0.112, 0.042, 0.092, 0.040, and 0.299, respectively. All collinear genes in the OsHsp70 family had Ka/Ks ratios less than 1, indicating that these genes have undergone strong purifying selection pressure during evolution.

**Table 1:** Ka/Ks ratio of *OsHsp70* homologous genes

Gene1	Gene2	Ka	Ks	Ka/Ks
hsp70-20	hsp70-10	0.041	0.368	0.112
hsp70-20	hsp70-4	0.015	0.362	0.042
hsp70-16	hsp70-1	0.122	1.328	0.092
hsp70-30	hsp70-4	0.040	1.000	0.040
hsp70-30	hsp70-32	0.294	0.983	0.299

### Tissue-specific expression analysis and expression analysis of *OsHsp70s* under heat stress

**Fig. 4:**
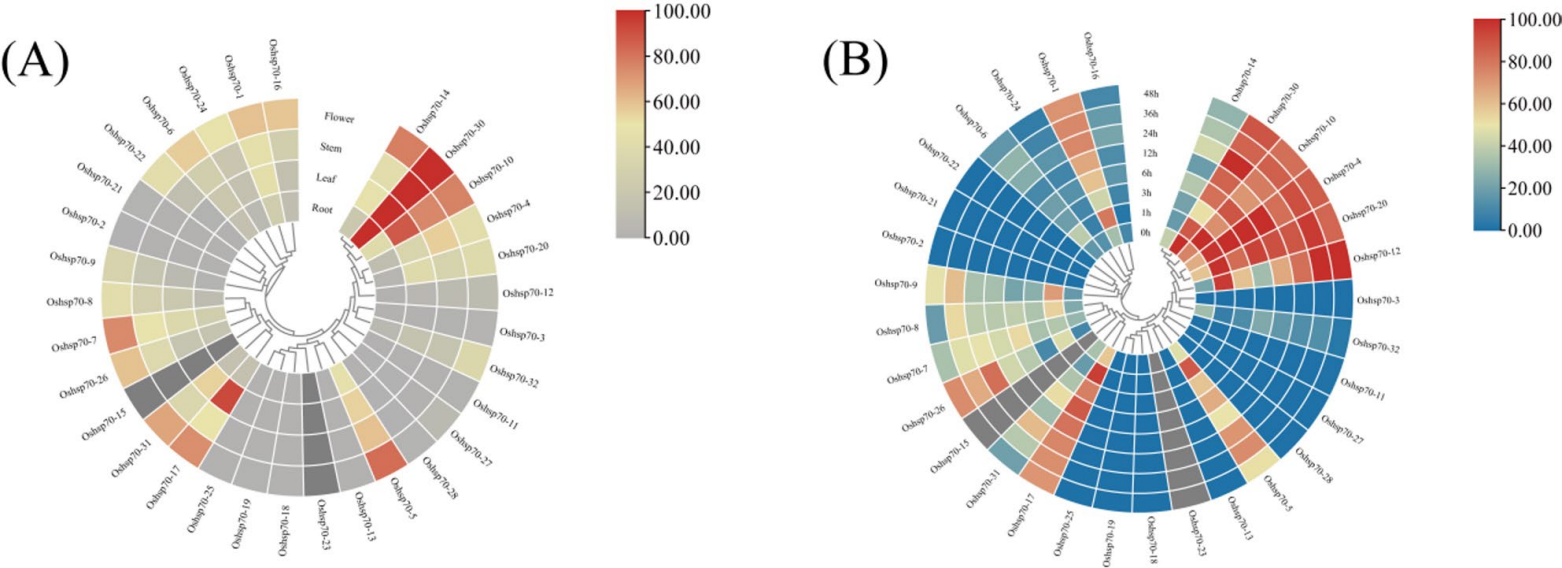
Expression analysis of *OsHsp70s*. **A** Analysis of tissue-specific expression of *OsHsp70s*. **B** Expression analysis of *OsHsp70s* under heat stress. The FPKM values represented by different colors in the figure are shown on the color bar on the right

The expression data of *OsHsp70* members in different tissues and under heat stress were obtained from the Rice RNA-seq Database, and visualized as heatmaps generated by TBtools software (Fig. 4). *OsHsp70s* were expressed in most tissues, and there were significant variations in the expression levels of different members within the same tissue. The tissue-specificity index (TAU) of the FPKM values for *OsHsp70* members was calculated, and genes with high expression levels were screened based on a threshold range of 0.15–0.85. A total of 17 *OsHsp70* members with high expression levels were identified, which may be utilized as candidate genes for research related to heat stress.

### Haplotype analysis of *OsHsp70s*

**Fig. 5:**
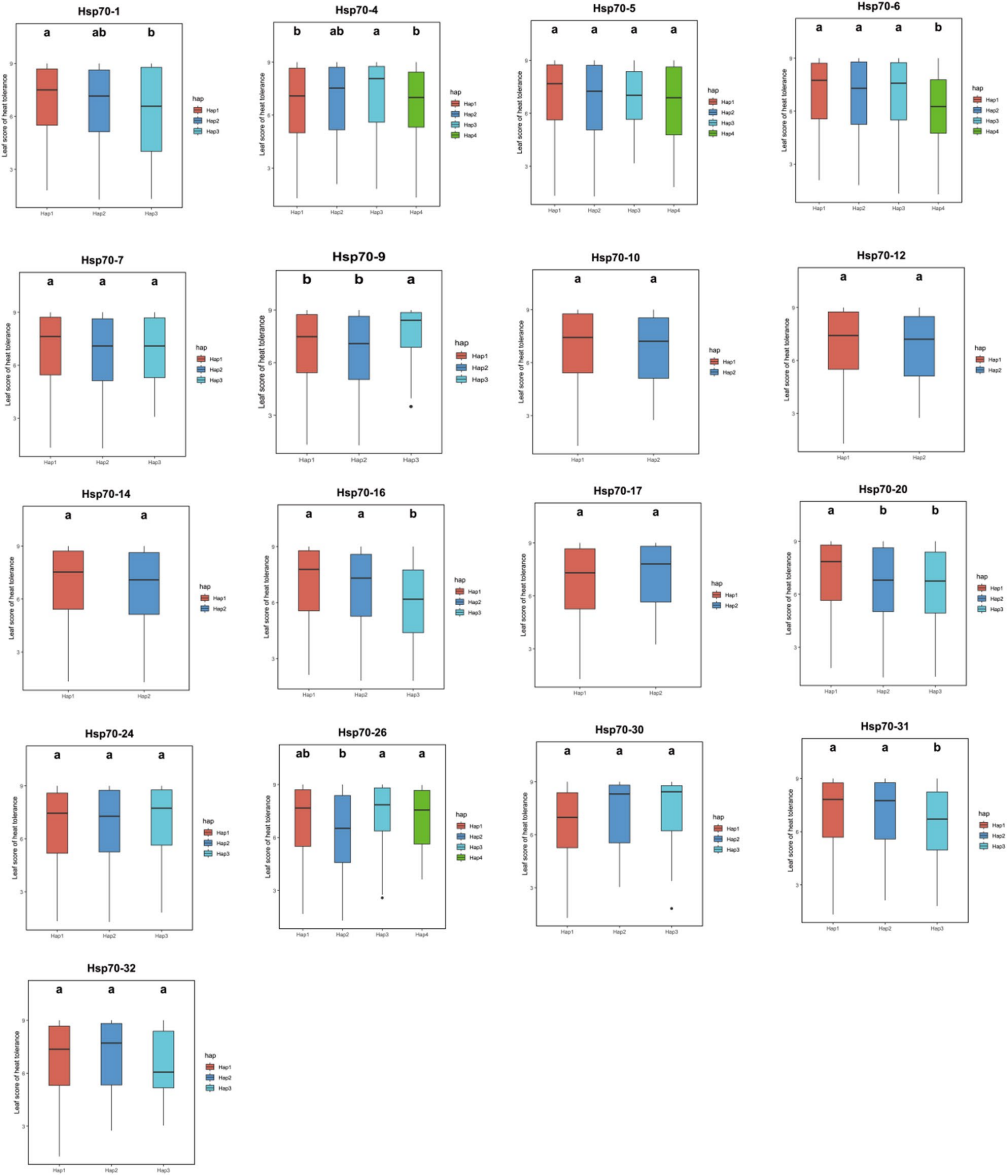
Haplotype analysis of 17 *OsHsp70s*. Different colors represent different haplotypes, and the letters in multiple comparison annotations indicate significant differences among the haplotypes

To further explore the relation of *OsHsp70s* with heat stress response, genotyping and phenotypic analyses were conducted on 17 *OsHsp70s* with high expression in rice (Fig. 5, Table S2). Among these genes, the haplotypes of only eight genes were genotyped,, including *OsHsp70-1*,* OsHsp70-4*,* OsHsp70-6*,* OsHsp70-9*,* OsHsp70-16*,* OsHsp70-20*,* OsHsp70-26*, and *OsHsp70-31.* Six of those genes exhibited significant differences(Table S3).

### Amino acid site mutations and prediction of deleterious mutation sites

**Fig. 6:**
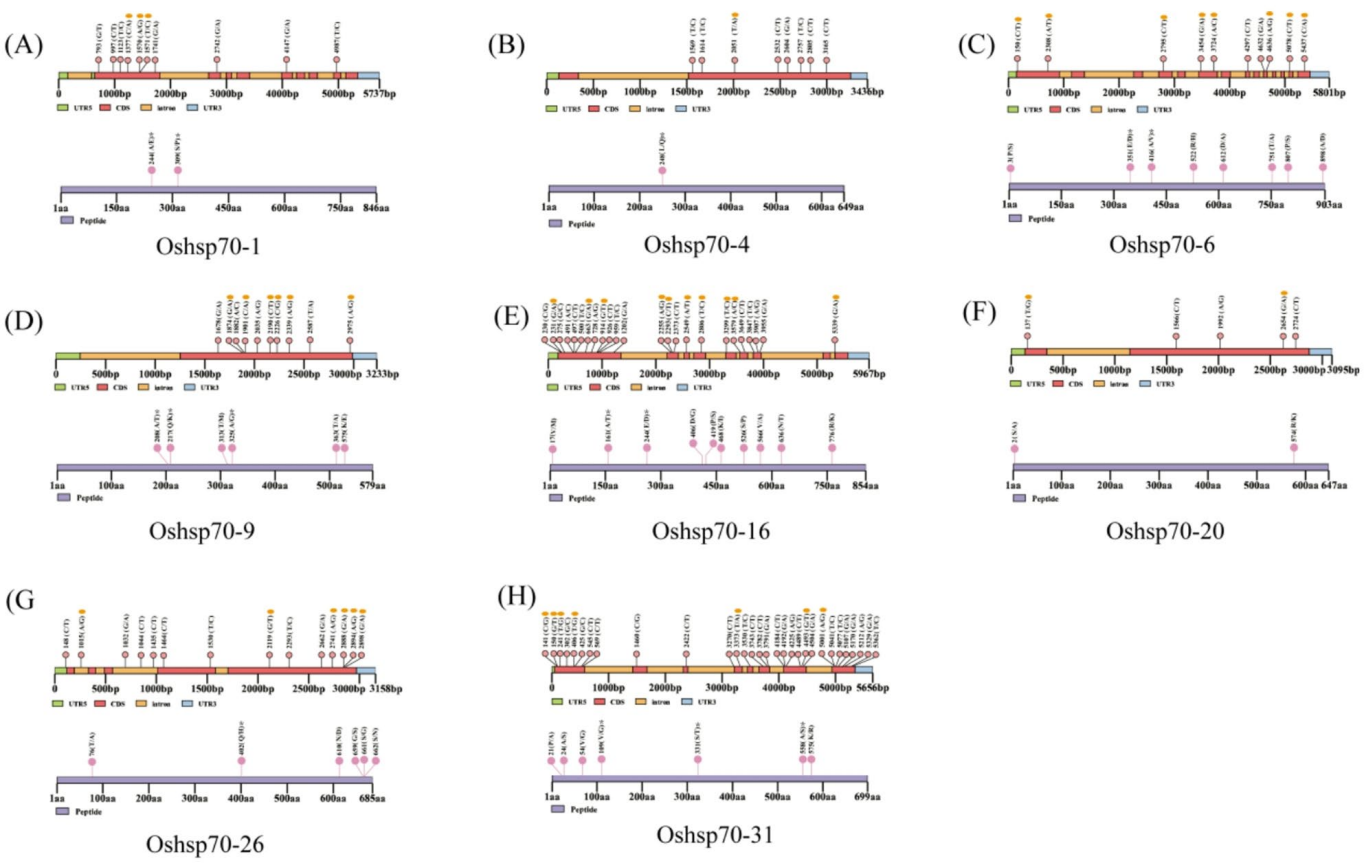
Mutation analysis of single-nucleotide polymorphism (SNP) sites in candidate OsHsp70 members.The 5' untranslated region (UTR5) sequences are represented by green squares, the coding DNA sequence (CDS) sequences by red squares, the intron sequences by yellow squares, the 3' untranslated region (UTR3) sequences by blue squares, and the amino-acid sequences by purple squares.The SNPs denoted by yellow ellipses are non-synonymous SNPs. The amino-acid substitutions indicated by five-pointed stars occur within the conserved motifs of the genes.

To further validate these candidate genes, we analyzed the mutation sites in the haplotypes with significant differences (Fig. 6). As a result, 10, 8, 10, 10, 24, 5, 14, and 30 nucleotide mutations were identified in *OsHsp70-1*,* OsHsp70-4*,* OsHsp70-6*,* OsHsp70-9*,* OsHsp70-16*,* OsHsp70-20*,* OsHsp70-26* and *OsHsp70-31*, among which 2, 1, 8, 6, 10, 2, 6, and 7 were non-synonymous mutations, respectively. Only 7 out of these 8 genes contained amino acid substitutions located on conserved motifs, namely OsHsp70-1, OsHsp70-4, OsHsp70-6, OsHsp70-9, OsHsp70-16, OsHsp70-26, and OsHsp70-31, with the corresponding numbers of substitutions being 2, 1, 2, 3, 2, 1, and 3 respectively.The findings of the deleterious-mutation prediction indicated that, among the eight genes, only three genes (hsp70-6, hsp70-9, hsp70-16) harbored deleterious-mutation sites, with the number of such sites being 1, 1, and 2 respectively (Table S4). Notably, the numbers of supporting sequences for the deleterious mutation sites of hsp70-6 and hsp70-16 were merely 65 and 177, respectively. In contrast, the number of supporting sequences for the deleterious mutation site of hsp70-9 was as high as 1493. This suggests that the amino-acid mutation from A to T at position 208 of its protein sequence is highly likely to exert a negative impact on the function of hsp70-9.

### Prediction of the secondary structure, domains, and conserved protein motifs of candidate *OsHsp70s*

**Fig. 7:**
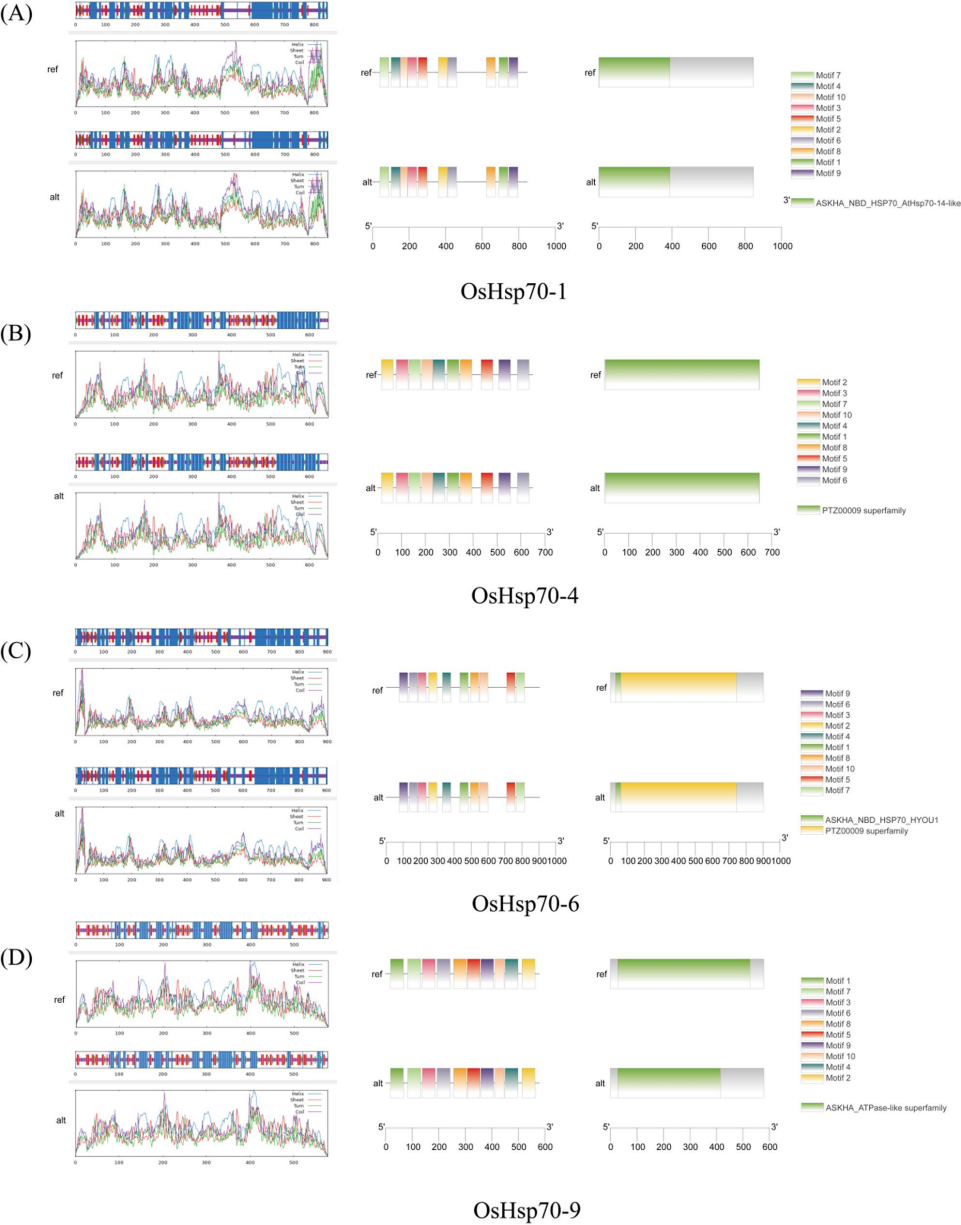
Prediction of the secondary structure, protein conserved motifs, and domain of candidate OsHsp70 members (ref), and after SNP site mutation (alt). The colored section on the left depicts the positions of the secondary structure within the corresponding amino-acid sequences. The squares of diverse colors in the middle signify different motifs. The green and yellow squares on the right denote the domains

The SOPMA online tool predicted that all eight candidate OsHsp70 proteins (OsHsp70-1, OsHsp70-4, OsHsp70-6, OsHsp70-9, OsHsp70-16, OsHsp70-20, OsHsp70-26 and OsHsp70-31) contained four basic secondary structure elements, including α-helix, β-sheet, β-turn, and random coil (Fig. 7, Table S5). Notably, the amino acid changes induced by SNPs led to obvious alterations in the composition of the secondary structures of these proteins. For example, the content of α-helix increased from 33.22% to 35.12% in OsHsp70-9, while it decreased from 45.82% to 42.41% in OsHsp70-20 after SNP induction. By predicting the protein domains of the candidate genes, we further investigated their functional differences. The results indicated that the mutation at the SNP site in the amino acid sequence of OsHsp70-9 led to changes in the domain. Therefore, based on the amino acid mutation, OsHsp70-9 was further confirmed as a potential key candidate gene for heat-stress tolerance in rice.

### Real-time fluorescent quantitative PCR analysis of *OsHsp70-9*

**Fig. 8:**
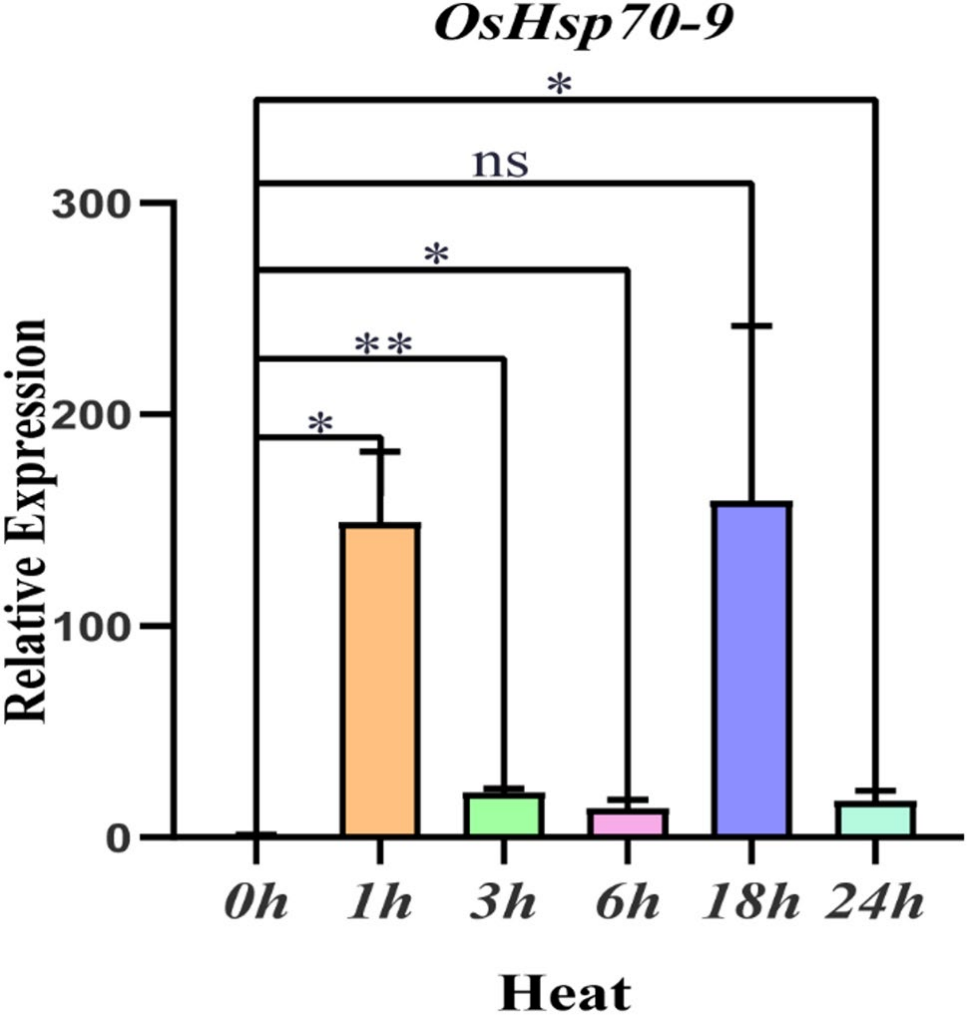
Expression analysis of the *OsHsp70-9* gene under heat stress. The data were statistically analyzed using WPS 2023 software, and analysis of variance was performed using IBM SPSS Statistics 25 software. The significance levels were defined as *** *p* < 0.001, ** *p* < 0.01, * *p* < 0.05

The relative gene expression of *OsHsp70-9* under heat stress was analyzed by quantitative reverse transcription polymerase chain reaction (qRT-PCR). After heat-stress, the expression of OsHsp70-9 was strongly induced, with a log₂FC range of 3.70–7.32. As shown in Fig. 8, the expression level of *OsHsp70-9* increased significantly after 1 h of heat stress, and exhibited a dynamically changing pattern with the prolonged heat stress time, indicating that it is responsive to heat stress and is a key candidate gene for heat stress tolerance in the *OsHsp70* family.

## Discussion

### Relationship between *OsHsp70* expression under heat stress and gene conservation

Gene expression is a fundamental process in all organisms, bridging the gap between the genetic material and functional biology. As the initial products in the hierarchical regulation of gene activity, mRNAs are generated under transcriptional regulation and degraded through RNA decay mechanisms. Therefore, in numerous genome-wide studies, the RNA level has been used as a direct indicator of gene activity [6, 26, 35]. The RNA level of a gene can also indicate the magnitude of selection pressure on amino acid substitution [11, 30]. There were five gene pairs with intragenic collinearity in the *OsHsp70* family, which involved *OsHsp70-20*,* OsHsp70-10*,* OsHsp70-4*,* OsHsp70-16*,* OsHsp70-1*,* OsHsp70-30*, and *OsHsp70-32*. Ka/Ks ratio analysis indicated that these genes have undergone strong purifying selection pressure, leading to the conservation of their functions. The Ka/Ks value of the gene pair hsp70-30/hsp70-32 indicates a relatively more permissive selection pressure compared to that of the other four pairs. This is due to the occurrence of a greater number of non - synonymous mutations between this pair of genes. Among them, five genes (*OsHsp*70-20, *OsHsp*70-10, *OsHsp*70-4, *OsHsp*70-1, and *OsHsp*70-30) showed relatively high expression levels under heat stress. Except for *OsHsp70-1*, all these genes belong to Group Ⅶ in the phylogenetic tree, indicating that genes with similar expression patterns also maintain similarities in their evolution.

### Effects of amino acid variations induced by SNPs on the secondary structure of *OsHsp70-9*

The haplotype of a gene generally refers to diverse combinations of variations in its promoter, 5’-untranslated region, coding region, and 3’-untranslated region. The haplotypes that are beneficial to humans are termed elite haplotypes. For example, introgression of the elite OsIRO2 - H3 haplotype into high - performing rice cultivars resulted in a 25.0%–27.3% increase in yield compared with the recurrent parent [38]. Liu et al. discovered that Minghui 63 has haplotype combination 4 (HC4), a superior thousand-grain weight (TGW) haplotype combination, which has a much higher TGW than its sister line without HC4, indicating that TGW-HC4 is an elite haplotype combination for TGW [24]. Another study identified haplotype combinations (HCs) through genetic variation analysis of 417 rice germplasms from different regions, where HC21 and HC24 exhibited extremely high UV-B tolerance, providing potential targets for molecular breeding aimed at developing UV-B-resistant rice varieties [49]. Amino acid sequences are the basis of protein structure and function, and their changes can affect protein function in various ways. Amino acid point mutations can be classified into synonymous and non-synonymous mutations. Synonymous mutations have no obvious impacts on amino acid sequences, while non-synonymous mutations may alter the three-dimensional structure of the protein, affect the configuration of the active site, and change protein-protein interactions, thereby modifying the functions of the protein. Zhong et al. reported that the SNP1 mutation changed methionine (wild-type) to threonine (mutant-type), and found that a single-base substitution (A to C) is the main reason for increased inducibility in maize [56]. In this study, we analyzed eight candidate genes (*OsHsp70-1*,* OsHsp70-4*,* OsHsp70-6*,* OsHsp70-9*,* OsHsp70-16*,* OsHsp70-20*,* OsHsp70-26* and *OsHsp70-31*), and found that these *OsHsp70* genes all have SNP variations and amino acid substitutions. Zhu et al. found that the elevated proportions of α - helix, β - turn, and random coil, coupled with the diminished levels of β - sheet, consistently led to a reduction in the average particle size [57]. Research into plant peptides has disclosed that peptides exhibit structural plasticity, alternating between α-helix and β-sheet conformations contingent upon their environment, a phenomenon that directly impacts their bioactivity [4]. Consequently, alterations in the secondary structure of plant proteins are also likely to exert substantial impacts on plant functions. Predictions on the secondary structures of the proteins encoded by these eight genes showed that they all contain four basic structural elements of α-helix, β-sheet, β-turn, and random coil. Notably, the amino acid variations induced by SNPs led to changes in the secondary structures of corresponding OsHsp70 proteins. These SNP-mediated structural variations, particularly the significant increase in α-helix content in OsHsp70-9 and decrease in OsHsp70-20, suggest potential impacts of these changes in protein secondary structure on the stability of protein folding and functional interactions. These structural variations may affect the relative proportion and spatial arrangement of the second structural elements, which may lead to functional divergence of the encoded proteins. Prediction on the conserved protein domains of these eight OsHsp70 members and their mutants showed that the mutation changed the domain of OsHsp70-9, indicating that mutation at the SNP site may affect the function of OsHsp70-9.

## Materials and methods

### Identification of the *OsHsp70* family members and phylogenetic analysis

The Hidden Markov Model (HMM) of OsHsp70 (PF00012) was downloaded from the Pfam database (Released in November 2019, Version 3.3). HMMER 3.0 was used to query the rice amino acid sequences with an E-value threshold of < 1E − 15 to identify the members of the *OsHsp70* family [53]. All DNA files and genome annotation files(GFF) were downloaded from the Ensembl database (Oryza_sativa.IRGSP − 1.0.62, accessed on July 17, 2025). Alternative splicing isoforms have been filtered. Both redundant sequences and truncated sequences have been excluded. To characterize the functional domains of candidate genes, we performed domain architecture analysis using PfamScan (Table S6). A phylogenetic tree was constructed using the Maximum Likelihood (ML) method in TBtools, with the following parameters: pairwise deletion, LG model, Four-category discrete gamma distribution, and Bootstrap value set to 5000. Only those stable branches exhibiting a bootstrap support value of at least 70% were retained as valid subgroups. Sub-family classification of the phylogenetic tree was carried out based on evolutionary distances. Then, the online mapping site iTOL (accessed May 26th, 2025; https://itol.embl.de/) was used to beautify the phylogenetic tree [20].

### *Cis*-acting element and collinearity analysis

The 2000-bp fragment upstream of *OsHsp70s* was taken as the promoter sequence, and the *cis*-acting elements in the promoter region were analyzed using PlantCARE [19]Accessed on September 14, 2025. Model Version: 1). The collinearity of duplicate gene pairs among *OsHsp70* family members and in different species (*Oryza sativa*,* Populus trichocarpa*,* Arabidopsis thaliana*,* Solanum lycopersicum*,* Cucumis sativus*,* Citrullus lanatus*,* Glycine max*,* Hordeum vulgare*,* Triticum aestivum*,* Zea mays*, and *Setaria italica*) was analyzed using MCScanX [45]E-value:1e-10, Num of BlastHits:5). The results were visualized using TBtools [8]. Non-synonymous (Ka) substitution and synonymous (Ks) substitution were calculated for each duplicated *OsHsp70* gene using KaKs_Calculator [54](Nei-Gojobori method).

### Tissue-specific expression and expression data of *OsHsp70s* under heat stress

The expression data of *OsHsp70s* in various tissues during rice growth and development and under heat stress were downloaded from the Rice RNA-seq database (as FPKM values, Transcripts per kilobase read per million maps; https://plantrnadb.com/ricerna/) [47]. After standardizing the expression data with GraphPad Prism, a heatmap was generated by means of TBtools [8]. The values within the heatmap represent the normalized frequency. The TAU index (tissue specificity index) of the FPKM values of the OsHsp70s members in rice was calculated using the TAU Calc tool in the R programming language, with a threshold range set between 0.15 and 0.85, to filter the tissue-specific levels of expression of the *OsHsp70* genes in rice [46].

### Heat stress treatment and qRT-PCR

At the Crop Molecular Breeding Innovation Center of Anhui Agricultural University, the rice seeds (Nipponbare) were disinfected with 3% sodium hypochlorite for 30 min, germinated at 28 °C for 3 days, and then transplanted into hydroponic boxes filled with Hoagland nutrient solution. Rice seedlings were randomly and thoroughly distributed in black 96-well culture boxes and allowed to grow for 14 days. Three biological replicates along with technical replicates were established. The plants were placed in an intelligent light-temperature incubator under normal conditions (28 °C for 12 h during the day, 28 °C for 12 h at night, 75% humidity, and light intensity of 20,000 lx: LED). The seedlings were subjected to a high-temperature treatment of 42 °C under the condition of 12-hour light/12-hour dark alternation. Rice leaves were collected at 0, 1, 3, 6, 18, and 24 h, immediately placed in liquid nitrogen, and stored at − 80 °C for total RNA extraction.

The collected samples were ground in liquid nitrogen using a mortar and pestle. The TaKaRa MiniBEST Plant RNA Extraction Kit was employed to extract RNA. The obtained cDNA was reverse transcribed using the TAKARA Reverse Transcription Kit. Gene expression was analyzed via qRT-PCR. Primers for the selected *OsHsp70-9* were designed. The relative quantitative expression was normalized to that of the reference gene *OsActin1* (LOC-Os03g61970) [9] (Table S7). Real-time fluorescence quantitative detection was performed using a LightCyler96 quantitative PCR instrument. The amplification system had a volume of 20 µL, with 2 µL of cDNA, 0.8 µL each of forward and reverse primers, 10 µL of AceQ Universal SYBR qPCR Premix, and 6.4 µL of ddH2O. The program parameters were configured as follows: Pre-denaturation was executed at 95 °C for 30 s. Subsequently, denaturation at 95 °C for 10 s and annealing-extension at 60 °C for 30 s were iterated for a total of 40 cycles.Regarding the melting curve analysis, the initial conditions were set at 95 °C for 15 s and 60 °C for 60 s. This was followed by raising the temperature to 95 °C at the instrument’s default rate and sustaining this temperature for 15 s. Fluorescence signals were continuously monitored throughout the entire process. Finally, the reaction was terminated by incubating at 37 °C for 30 s [51]. The 2^−ΔΔCt^ method was used to analyze the expression of each gene [41]. The WPS2023 software was used for statistical analysis of the data, and the GraphPad Prism8 software was used for analysis of variance and graphing.

### Haplotype analysis of *OsHsp70s* and protein site mutations

The gcHap data of OsDLH genes was downloaded from the RFGB (rmbreeding.cn) on April 7, 2025. For SNP detection, the BWA-MEM (0.7.10) was used to align with the Nipponbare reference genome. After sorting and duplicate removal by Picard (1.119) and Indel region correction by GATK (3.2-2), single-sample detection was carried out through GATK UnifiedGenotyper, and then genotyping was completed by combining 3010 VCF files. The filtering steps were as follows: first, retain the loci supported by ≥ 1 sample and with QUAL ≥ 30. In the basic set, non - biallelic SNPs with abnormal heterozygosity were removed. For the filtered set, the missing call rate was required to be ≤ 20% and the minor allele frequency (MAF) ≥ 1%. In the core set, redundancy was removed through two-step LD pruning (r²≤0.8) by PLINK [43].The haplotype analysis of *OsHsp70* family members was conducted using the heat-tolerance phenotype data of 620 rice germplasms from natural populations. The heat-tolerant phenotype data were retrieved from the research conducted by Li et al. [22]. Li et al. subjected 13-day-old seedlings to a 3-day treatment at 45 °C, after which the seedlings underwent a 7-day recovery under normal conditions at 28 °C. Additionally, they measured phenotypic data, including the leaf heat-tolerance score (SHT). The Leaf Heat-Tolerance Score (SHT) is a scoring system that employs a 1-9-point scale to quantitatively evaluate the phenotypic damage of plant seedlings under heat stress. The scores are associated with the level of seedling damage, increasing in sequence from abnormal leaf-tip morphology, through various degrees of leaf desiccation, to the death of the entire plant. SnapGene (version 6.0.2) was used to analyze whether mutations occur at the nucleotide and amino acid sites of *OsHsp70* members, and the R package (lollipop plot) was used to visualize the *OsHsp70* members in the amino acid site mutations. The conserved domains of OsHsp70 proteins were queried through the NCBI Protein Batch CD-search database [29]. Conserved motifs in the OsHsp70 protein sequences were analyzed using MEME, with the maximum number of motifs set to 10 [5]. TBtools was used to predict the conserved protein domains of candidate OsHsp70 members and their mutant variations. The PROVEAN tool was utilized to predict whether non-synonymous mutations would be classified as deleterious mutations(accessed November 30th, 2025;http://provean.jcvi.org/seq_submit.php).

## Supplementary Information

Supplementary Material 1.

Supplementary Material 2.

## Data Availability

Generated Statement: The original contributions presented in the study are included in the article/supplementary material, further inquiries can be directed to the corresponding author/s.
