# Supplementary material for: Genome-wide analysis of the Hsp70 gene family in rice reveals that OsHsp70-9 plays a significant role in heat stress response
Source: BMC Plant Biol. 2025 Dec 28;26:303. doi: 10.1186/s12870-025-07975-9 (PMC12903685; doi:10.1186/s12870-025-07975-9)
Supplement: Supplementary file 2 — Supplementary Material 2. [file 12870_2025_7975_MOESM2_ESM.docx]

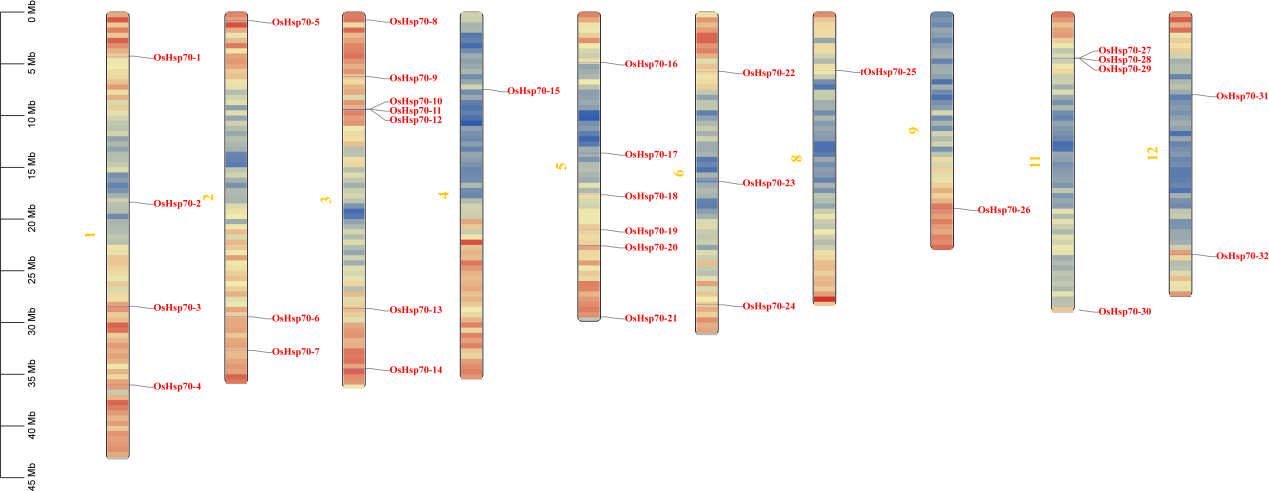


Figure.S1 Chromosomal localization of Oshsp70s.


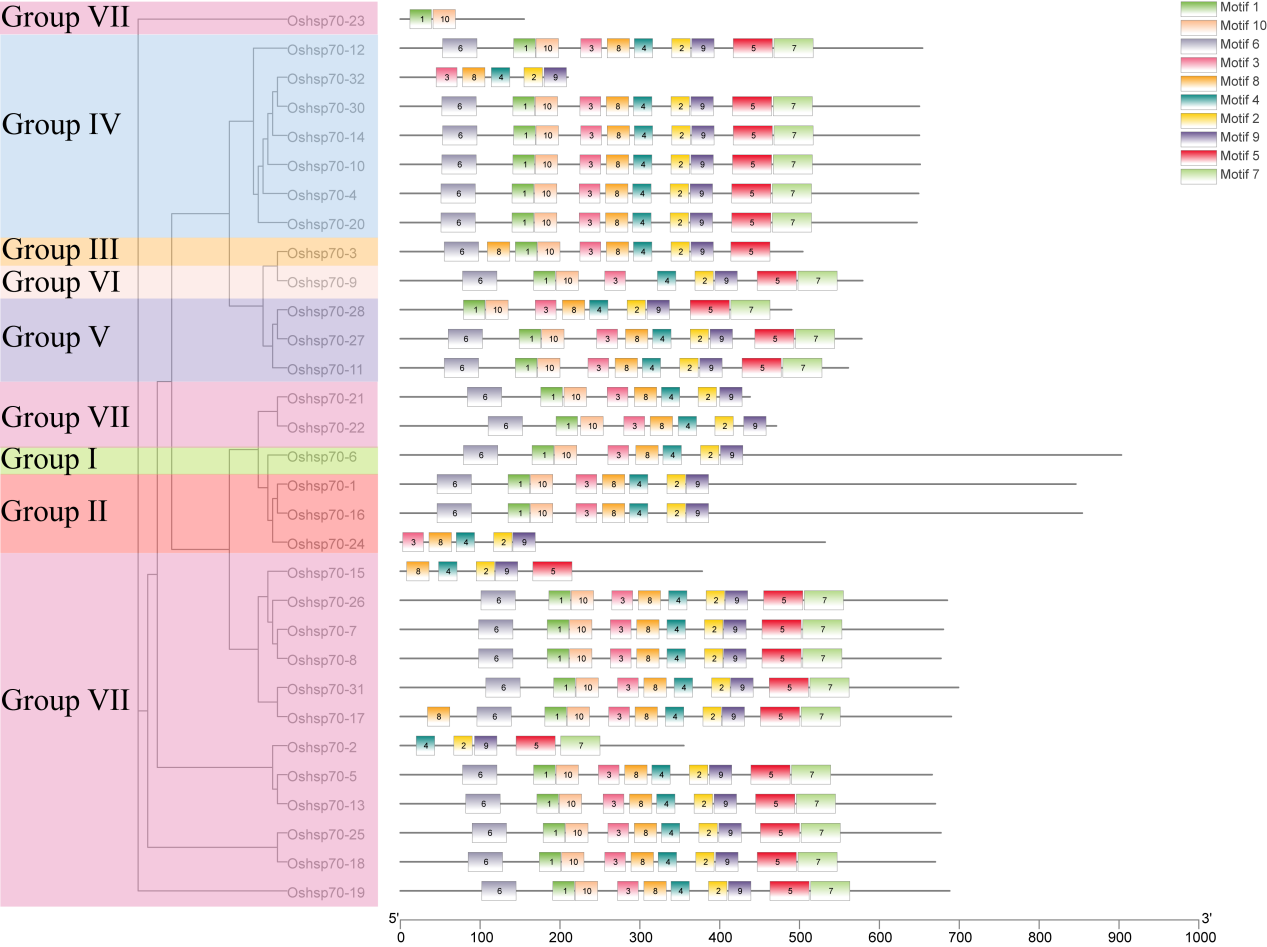


Figure.S2 Phylogenetic tree, motif prediction of Oshsp70s from left to right.
